# Emerging roles of mitochondrial sirtuin SIRT5 in succinylation modification and cancer development

**DOI:** 10.3389/fimmu.2025.1531246

**Published:** 2025-01-29

**Authors:** Zhangmin Ke, Kaikai Shen, Li Wang, Hao Xu, Xia Pan, Zhenjue Qian, Yuting Wen, Tangfeng Lv, Xiuwei Zhang, Yong Song

**Affiliations:** ^1^ Department of Respiratory and Critical Care Medicine, Affiliated Jiangning Hospital of Nanjing Medicine University, Nanjing, China; ^2^ Department of Respiratory and Critical Care Medicine, Jinling Hospital, Nanjing Medical University, Nanjing, China; ^3^ Department of Respiratory and Critical Care Medicine, Jinling Hospital, Affiliated Hospital of Medical School, Nanjing University, Nanjing, China; ^4^ Department of Respiratory and Critical Care Medicine, The People’s Hospital of Danyang, Affiliated Danyang Hospital of Nantong University, Zhenjiang, China

**Keywords:** SIRT5, cancer, succinylation, desuccinylase, sirtuin

## Abstract

Succinylation represents an emerging class of post-translational modifications (PTMs), characterized by the enzymatic or non-enzymatic transfer of a negatively charged four-carbon succinyl group to the ϵ-amino group of lysine residues, mediated by succinyl-coenzyme A. Recent studies have highlighted the involvement of succinylation in various diseases, particularly cancer progression. Sirtuin 5 (SIRT5), a member of the sirtuin family, has been extensively studied for its robust desuccinylase activity, alongside its deacetylase function. To date, only a limited number of SIRT5 substrates have been identified. These substrates mediate diverse physiological processes such as glucose oxidation, fatty acid oxidation, ammonia detoxification, reactive oxygen species scavenging, anti-apoptosis, and inflammatory responses. The regulation of these activities can occur through either the same enzymatic activity acting on different substrates or distinct enzymatic activities targeting the same substrate. Aberrant expression of SIRT5 has been closely linked to tumorigenesis and disease progression; however, its role remains controversial. SIRT5 exhibits dual functionalities: it can promote tumor proliferation, metastasis, drug resistance, and metabolic reprogramming, thereby acting as an oncogene; conversely, it can also inhibit tumor cell growth and induce apoptosis, functioning as a tumor suppressor gene. This review aims to provide a comprehensive overview of the current research status of SIRT5. We discuss its structural characteristics and regulatory mechanisms, compare its functions with other sirtuin family members, and elucidate the mechanisms regulating SIRT5 activity. Specifically, we focus on the role of succinylation modification mediated by SIRT5 in tumor progression, highlighting how desuccinylation by SIRT5 modulates tumor development and delineating the underlying mechanisms involved.

## 1 Introduction

Cancer continues to pose a significant global health challenge. According to recent data, the countries with the highest incidence of new cancer cases include China, the United States, India, Japan, Germany, Brazil, Russia, France, the United Kingdom, and Italy (1–3). Emerging research underscores the critical role of PTMs in tumor progression (4, 5). a member of the sirtuin family, was initially characterized as a mitochondrial deacetylase but has since been recognized for its diverse roles in PTMs, particularly desuccinylation (6). Located on chromosome locus 6p23, SIRT5 is an NAD-dependent deacetylase that contains a Zn²^+^ binding domain and a Rossmann fold domain, which together form the substrate binding site and the NAD^+^ binding site (6). Compared to other sirtuins, SIRT5 features a larger lysine acyl-binding pocket, resulting in relatively weaker deacetylase activity but robust desuccinylase activity (7). SIRT5 stands out among sirtuins due to its unique ability to remove succinyl groups from lysine residues, significantly influencing protein function and cellular metabolism (8). The biological significance of desuccinylation has garnered increasing attention, especially in the context of cancer. By modulating the succinylation status of key metabolic enzymes, SIRT5 affects various cellular processes, including glycolysis, mitochondrial function, and cell proliferation (9–11). This modulation is crucial because succinylation can either activate or inhibit enzymes involved in these pathways, thereby impacting tumorigenesis and cancer progression.

PTMs encompass a diverse array of chemical alterations where modifying groups covalently bind to substrate proteins, thereby altering their physiological properties such as activity, cellular localization, stability, and interactions with other proteins, ultimately influencing their function (12). Advances in proteomics have led to the identification of various PTM types, including acetylation, propionylation, methylation, butyrylation, succinylation, crotonylation, malonylation, ubiquitination, and 2-hydroxyisobutyrylation. As proteomics continues to advance, an increasing number of studies are reporting the involvement of PTMs in cancer development and progression (13, 14). Among these PTMs, succinylation has garnered significant attention in recent years for its role in tumorigenesis. Succinylation involves the reversible and dynamic covalent attachment of a succinyl group (-COCH_2_-CH_2_-COOH), donated by succinyl-CoA, to amino acid residues, predominantly lysine, within substrate proteins (15). This process is evolutionarily conserved and plays a critical role in numerous biological processes. Aberrant lysine succinylation has been shown to significantly impact metabolic pathways, gene transcription, DNA damage responses, and protein folding, stability, and functionality (16). Although several studies have documented the involvement of succinylation in various physiological and pathological processes (17, 18), including tumor biology (19), elucidating the regulatory mechanisms underlying succinylation in cancer can provide novel insights for prevention and therapeutic strategies.

In summary, the research surrounding SIRT5 and its desuccinylation activity underscores its pivotal role in cancer biology. By modulating the succinylation status of key metabolic enzymes and interacting with tumor suppressor pathways, SIRT5 exerts multifaceted influences on tumorigenesis. The ongoing elucidation of SIRT5’s functions and mechanisms may pave the way for novel therapeutic approaches that target metabolic pathways in cancer treatment, potentially improving outcomes for patients with malignancies characterized by dysregulated metabolic processes (20, 21). Therefore, this review aims to provide a comprehensive overview of the current research status of SIRT5. Specifically, it seeks to elucidate the role of SIRT5-mediated succinylation modification in tumors, thereby establishing a theoretical foundation for understanding the mechanisms of tumor development. Additionally, this review offers new perspectives for tumor therapy and drug development, highlighting the potential of targeting SIRT5 and succinylation as innovative strategies for combating cancer.

## 2 SIRT5

### 2.1 Structure and functional characteristics of SIRT5

The human SIRT5 gene, located at chromosome locus 6p23, encodes two protein isoforms comprising 310 and 299 amino acids, respectively. Predominantly localized within the mitochondria, with minor presence in the cytoplasm, SIRT5 exhibits a complex structural architecture. The protein consists of 14 β-strands and 9 α-helices that form both the zinc-binding domain and the Rossmann fold domain, thereby creating the substrate and NAD^+^ binding sites (6). Within the substrate binding site, three hydrophobic residues—phenylalanine-223 (Phe223), leucine-227 (Leu227), and valine-254 (Val254)—form the entrance for acyl-lysine groups. Two non-hydrophobic residues, tyrosine-102 (Tyr102) and arginine-105 (Arg105), specifically recognize the negatively charged acyl-lysine structure. Additionally, alanine-86 (Ala86) contributes to the formation of a larger lysine acyl-binding pocket in SIRT5 (22). These structural features confer SIRT5’s preference for short-chain carboxylates, such as malonyl, succinyl, and glutaryl groups, over acetyl groups (23). Consequently, the catalytic efficiency of SIRT5 for desuccinylation, demalonylation, and deglutarylation activities is approximately 1000-fold higher than its deacetylase activity (24).

### 2.2 Expression patterns and regulatory mechanisms of SIRT5

SIRT5 exhibits widespread expression across various organs, including the brain, heart, liver, kidneys, muscles, and testes, with predominant localization within mitochondria; however, it is also detectable in the cytoplasm and nucleus (25). In mammals, SIRT5 functions as a primary regulator of lysine desuccinylation. In mouse liver and embryonic fibroblasts, a comprehensive proteomic analysis identified 2,565 succinylation sites across 779 proteins. Upon SIRT5 gene knockout, over 90% of these sites demonstrated increased succinylation levels, primarily affecting proteins involved in the tricarboxylic acid (TCA) cycle and fatty acid metabolism (26). Consistent with these findings, Rardin et al. (27) reported that in the absence of SIRT5, 386 succinylation sites across 140 proteins in mouse liver mitochondria exhibited enhanced succinylation. These succinylated proteins predominantly participate in energy metabolism, β-oxidation, and ketone body production. Recent studies have further elucidated the role of SIRT5 in cardiac tissues, identifying key targets that suggest SIRT5-mediated deglutarylation may play a crucial role in maintaining cardiac energy metabolism (28, 29). This evidence underscores the importance of SIRT5 in regulating metabolic pathways critical for cellular function and homeostasis.

Despite the identification of numerous SIRT5 substrates, including a variety of metabolic enzymes, research into its desuccinylase, demalonylase, and deglutarylase activities is still in its early stages. This relative paucity of research can be attributed to several factors. Firstly, the discovery of SIRT5’s non-acetylation-related enzymatic activities is relatively recent compared to its deacetylase function, which has been extensively studied over the years. The novel nature of these modifications means that specific tools and methodologies for their detection and study are still being developed, refined, and disseminated within the scientific community. Secondly, the complexity of succinylation, malonylation, and glutarylation as PTMs poses additional challenges. These PTMs occur at lower abundances than acetylation and require highly sensitive and specific analytical techniques, such as mass spectrometry coupled with enrichment strategies, for reliable detection and quantification. The technical hurdles associated with studying these modifications have likely slowed progress in this area. Furthermore, the functional significance of these PTMs is not yet fully understood, which may lead to a lack of targeted research efforts. While it is clear that they play critical roles in cellular metabolism and other biological processes, the exact mechanisms by which they influence protein function and cellular physiology remain to be elucidated. As the importance of these modifications becomes more apparent, interest and investment in this field are expected to increase, driving further discoveries. Continued advancements in technology, along with growing awareness of the importance of these PTMs, are likely to facilitate deeper exploration and understanding of SIRT5’s role in regulating these modifications and their implications for health and disease. In summary, while significant strides have been made in identifying SIRT5 substrates, the full spectrum of its enzymatic activities, particularly those related to desuccinylation, demalonylation, and deglutarylation, remains to be thoroughly investigated. Addressing these knowledge gaps will be crucial for advancing our understanding of SIRT5’s functions and developing potential therapeutic targets for diseases characterized by dysregulated metabolic processes.

### 2.3 Comparison of SIRT5 with other members of the sirtuin family

In addition to SIRT5, the sirtuin family encompasses proteins with distinct characteristics and structures. The sirtuins constitute a highly conserved family of proteins, comprising seven members in mammals (SIRT1-7), which regulate various metabolic and stress response pathways (30). Specifically, SIRT1 and SIRT2 are predominantly localized in the cytoplasm, while SIRT3, SIRT4, and SIRT5 reside within mitochondria, and SIRT6 and SIRT7 are found in the nucleus (31). These proteins play pivotal roles in genomic stability, cell cycle regulation, metabolism, aging, and disease development (32). Sirtuins possess NAD^+^-dependent deacetylase activity (SIRT1, SIRT2, SIRT3, SIRT5, SIRT6, and SIRT7) or mono-ADP-ribosyltransferase activity (SIRT4 and SIRT6). Each sirtuin exhibits distinct enzymatic activities, biological functions, and subcellular localizations, which contribute to their diverse roles in cancer biology (**Table 1**).

**Table 1:** Comparison of SIRT5 with other SIRTs.

Name	Length	Structure	Location	Enzyme activity	Oncogene	Anti-cancer
SIRT1	747	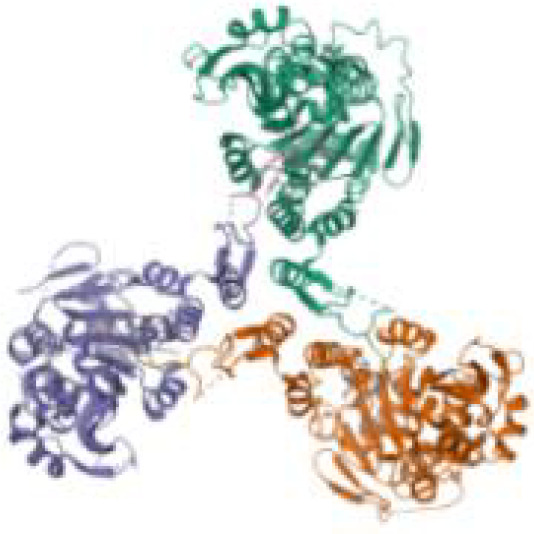	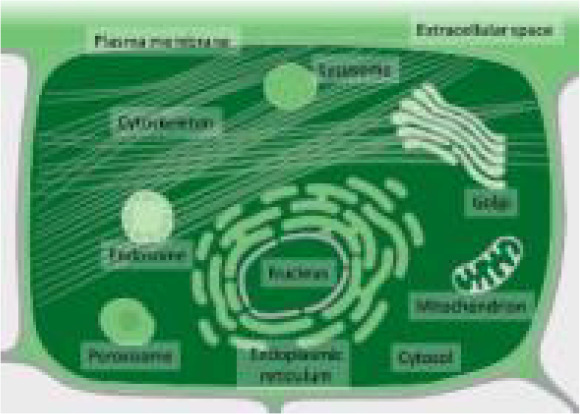	Deacetylase	BC (39), PC (74), CRC (38), LUD (75)	AML (76), GC (77)
SIRT2	389	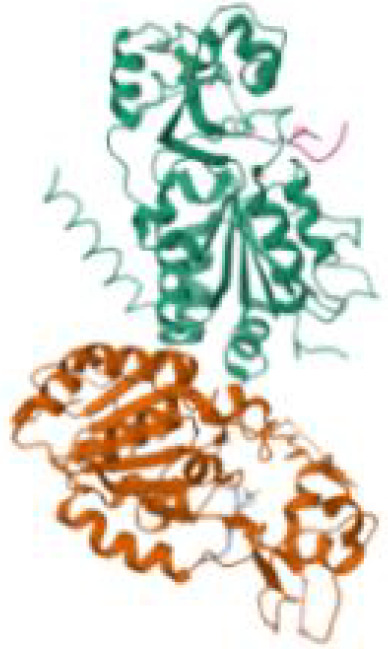	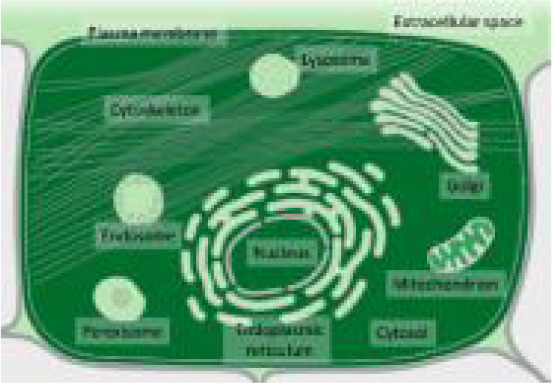	Deacetylase	AML (78), EC (79), BC (80)	BC (81), HCC (82)
SIRT3	399	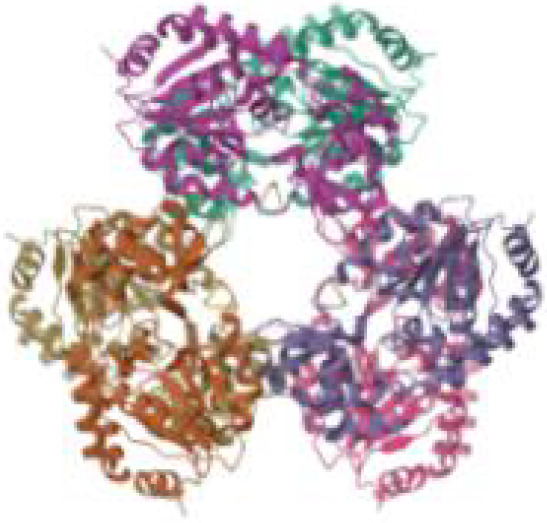	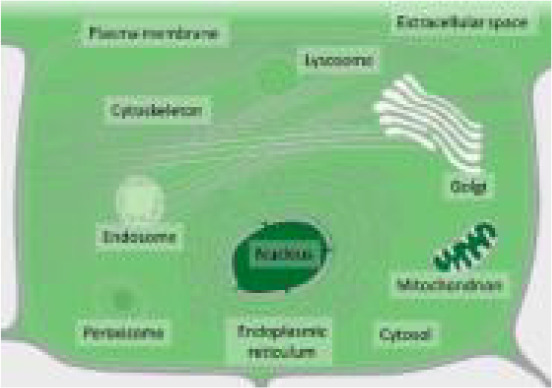	Deacetylase	GC (83), BC (84), CRC (85)	Glioma (86), OC (87), NSCLC (88), HCC (89)
SIRT4	314	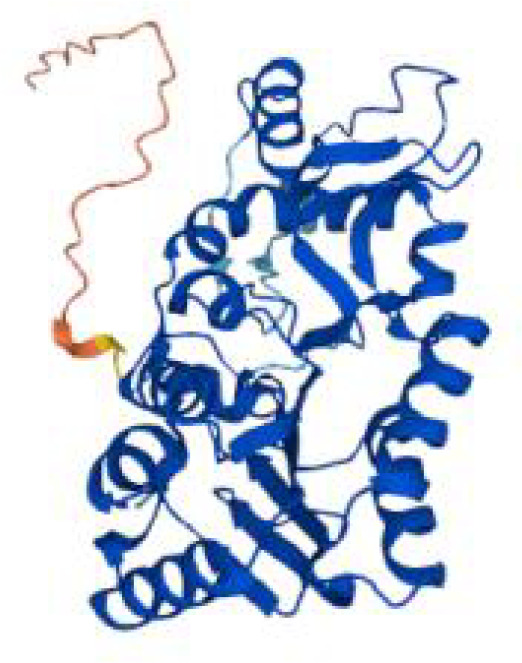	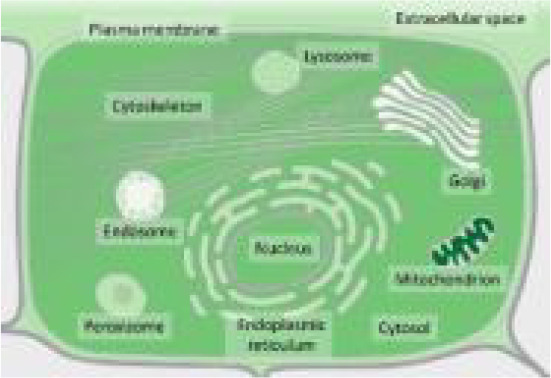	1. ADP ribosyltransferase 2. Lipoamidase 3. Deacetylase	HCC (90)	PC (60), GC (91), NSCLC (92), CRCC (93)
SIRT5	310	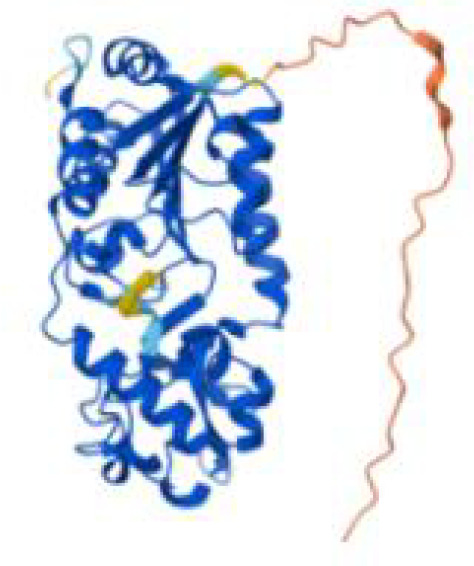	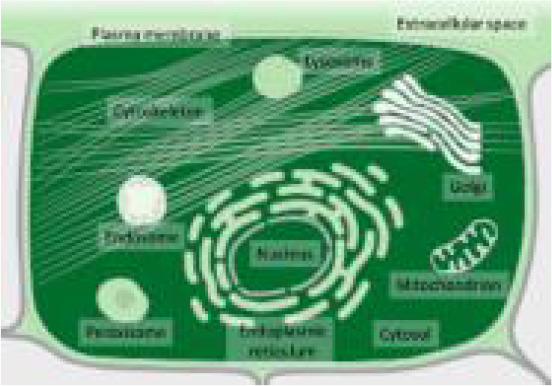	1. Succinyl deacylase 2. Malonyl deacylase 3. Deacetylase	CRC (94), NSCLC (95), PC (96)	HCC (97)
SIRT6	355	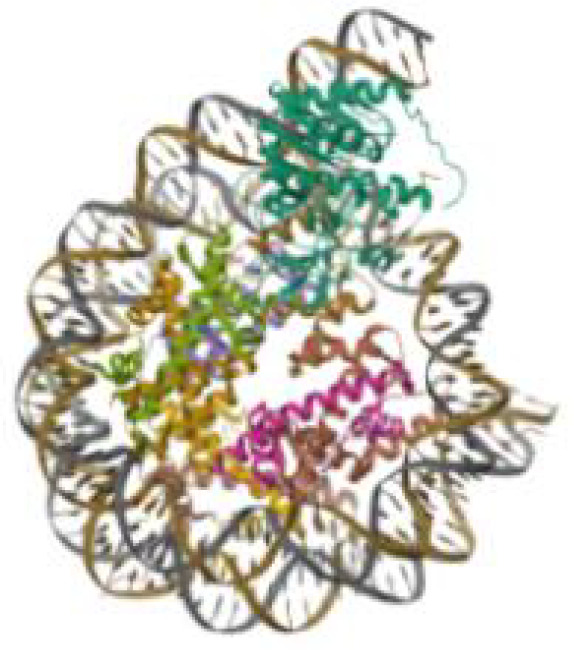	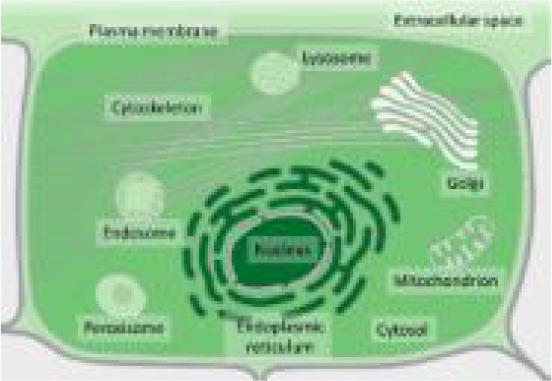	1. Deacetylase 2. ADP ribosyltransferase 3. Long-chain fattyacyl deacylase	HCC (98), PC (99), BC (100), MM (101), SCC (102),	Glioma (103), CRC (104), OC (105), NSCLC (106), HCC (107)
SIRT7	400	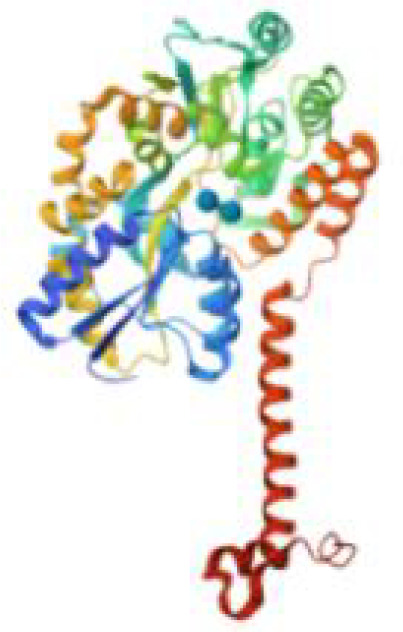	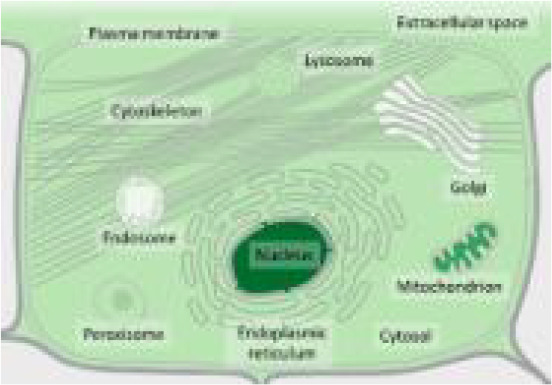	1. Succinyl deacylase 2. Deacetylase	HCC (108), GC (109), CRC (110), OC (111), CC (112)	BC (71)

BC, breast cancer; PC, prostate cancer; CRC, colorectal cancer; LUD, lung adenocarcinoma; AML, acute myeloid leukemia; EC, endometrial cancer; CC, cervical cancer. HCC, hepatocellular carcinoma; GC, gastric cancer; OC, ovarian cancer; NSCLC, non-small cell lung cancer; CRCC, chromophobe renal cell carcinoma; MM, multiple myeloma; SCC, squamous cell carcinoma; PDAC, pancreatic ductal adenocarcinoma.

For instance, SIRT1, an NAD^+^-dependent histone deacetylase belonging to the sirtuin family, has been closely associated with tumor development (33). It influences a wide array of processes, including cellular senescence (34), DNA replication (35), cell growth, metabolism (36), and tumor progression (37). Studies indicate that SIRT1 impacts the onset and progression of various cancers, such as colorectal, prostate, breast, and murine lung cancers, sarcomas, and lymphomas (38–40). Given its high expression in certain tumor tissues and its inhibition of several tumor suppressor genes like FOXO1, p73, and WRN (41–43), SIRT1 is believed to promote tumorigenesis. SIRT1 primarily modulates transcription factors, histones, and other non-histone substrates through deacetylation, thereby affecting gene expression patterns (35, 37). In contrast, SIRT5 exhibits a broader range of actions, encompassing not only deacetylation but also desuccinylation and demalonylation activities (27). While both SIRT1 and SIRT5 are involved in metabolic regulation, SIRT1 is more closely associated with nutrient-sensing signaling pathways, whereas SIRT5 focuses on the direct regulation of metabolic enzymes. This distinction highlights the specialized roles of each sirtuin in cellular metabolism and underscores the importance of understanding their individual contributions to cancer biology.

SIRT2, predominantly localized in the cytoplasm but also present in mitochondria and nuclei, deacetylates a variety of endogenous substrates, playing a significant role in multiple physiological and pathological processes. These include cancer cell proliferation, cell cycle regulation, apoptosis, genomic integrity, cellular metabolism, infection, and inflammation (44–46). Notably, SIRT2 exhibits both oncogenic and tumor-suppressive functions across different cancer types, indicating context-specific roles in cancer progression (47). While both SIRT5 and SIRT2 possess deacetylase activity, their distinct subcellular distributions determine their primary functions. The mitochondrial localization of SIRT5 positions it as a key regulator of metabolic processes, particularly in energy production and metabolite conversion (48). In contrast, SIRT2’s presence in the cytoplasm involves it more prominently in processes such as cytoskeletal dynamics, cell division, and signaling pathways (49).

SIRT3 is a critical mitochondrial deacetylase that plays an essential role in regulating protein acetylation levels, maintaining mitochondrial integrity, and modulating energy metabolism (50, 51). Hyper-acetylation, frequently observed in tumors, contributes to cancer survival by altering protein function. SIRT3 counteracts this hyper-modification, thereby modulating tumor progression (52). Moreover, SIRT3 can reprogram metabolism, significantly impacting tumor initiation and progression (53). However, its dual nature, exhibiting both pro- and anti-tumorigenic effects, complicates its targeting for therapeutic purposes (54, 55). Both SIRT3 and SIRT5 are principal sirtuin members within the mitochondria, each contributing uniquely to mitochondrial function. SIRT3 is renowned for its antioxidant effects and metabolic control, enhancing mitochondrial efficiency and reducing oxidative stress (56). It acts primarily as a deacetylase, normalizing hyper-acetylation and supporting mitochondrial health. Conversely, SIRT5 exhibits a broader range of demodification activities within the mitochondria. It plays a crucial role in fatty acid oxidation (28) and amino acid metabolism (57), including the regulation of arginase II activity via desuccinylation, which influences the urea cycle (58).

SIRT4 significantly inhibits glutamine metabolism by ADP-ribosylating glutamate dehydrogenase, thereby limiting the supply of energy and materials required for rapid proliferation in tumor cells (59, 60). This effect has been confirmed across various cancer types, including breast cancer (61), colorectal cancer (62), esophageal squamous cell carcinoma (63), and thyroid cancer (64). The consensus that SIRT4 suppresses tumor development through inhibition of glutamine metabolism suggests its potential as a novel biomarker and therapeutic target for malignancies. While both SIRT5 and SIRT4 act within mitochondria, they exhibit distinct functional orientations and mechanisms. SIRT5 primarily modulates metabolic enzyme activity through its unique demodification activities, whereas SIRT4 affects metabolic processes by regulating signaling pathways. Specifically, SIRT5’s enzymatic actions are more directly involved in the regulation of metabolic enzyme activity, while SIRT4 plays a more significant role in signal transduction and metabolic network regulation.

SIRT6 is predominantly localized in the nucleus and possesses two key enzymatic activities: NAD^+^-dependent deacetylase and mono-ADP-ribosyltransferase. These activities are integral to SIRT6’s functions (65). Studies have shown that, acting as a deacetylase for histone H3 lysine 9 (H3K9), SIRT6 controls the expression of various glycolytic genes, particularly by co-repressing the transcription factor hypoxia-inducible factor 1α (HIF-1α), thus inhibiting tumor progression (66). Moreover, overexpression of SIRT6 can induce apoptosis in fibrosarcoma and human cervical cancer cell lines via its mono-ADP-ribosyltransferase activity without affecting normal cells (67). Conversely, SIRT6 has also been shown to enhance cytokine secretion and cell motility, and increase drug resistance by hyperactivating calcium channels, playing a pro-oncogenic role (68). This dual action of SIRT6 appears to depend on tissue context, spatiotemporal distribution of various factors, and different stages of tumorigenesis. Despite sharing deacetylase activity, SIRT5 and SIRT6 differ significantly in their biological functions due to their distinct subcellular localizations. SIRT5 operates mainly within the mitochondria, influencing metabolic pathways and energy conversion, whereas SIRT6 is active in the nucleus, participating in DNA repair and gene expression regulation. This disparity underscores the Sirtuin family’s capability to perform diverse functions within the cell, contributing to cellular health and adaptation to environmental changes.

SIRT7, primarily localized in the nucleolus, has recently been identified as possessing deacetylase activity towards specific substrates, thereby influencing cellular life activities through various pathways (69). Overexpression of SIRT7 has been observed in several human malignancies, including hepatocellular carcinoma (70), breast cancer (71), thyroid cancer (72), gastric cancer (73), and others. Its expression levels correlate with clinical-pathological features and patient prognosis, underscoring its potential role in tumor biology. The oncogenic effects of SIRT7 are closely linked to its deacetylation activity, which primarily influences gene expression regulation. Unlike SIRT7, which predominantly affects transcriptional regulation, SIRT5 plays a crucial role in cellular energy metabolism. The mitochondrial localization of SIRT5 establishes it as a key regulator of metabolic enzyme activity, whereas the nuclear role of SIRT7 positions it as an important participant in gene expression modulation. These two sirtuin members influence cellular health through distinct mechanisms: SIRT5 by modulating metabolic pathways and SIRT7 by regulating gene expression.

In summary, while all members of the sirtuin family depend on NAD^+^ and can influence metabolic processes, each member exhibits specific cellular localization and functional characteristics. SIRT5 stands out for its importance in non-conventional lysine modifications, such as desuccinylation, which are less common among other sirtuin members. Additionally, the unique role of SIRT5 in mitochondrial metabolism, particularly in fatty acid oxidation, highlights its significance in cellular energy production and tumor progression.

## 3 The succinylation modification mechanism regulated by SIRT5

### 3.1 Definition of succinylation modification

**Figure 1:**
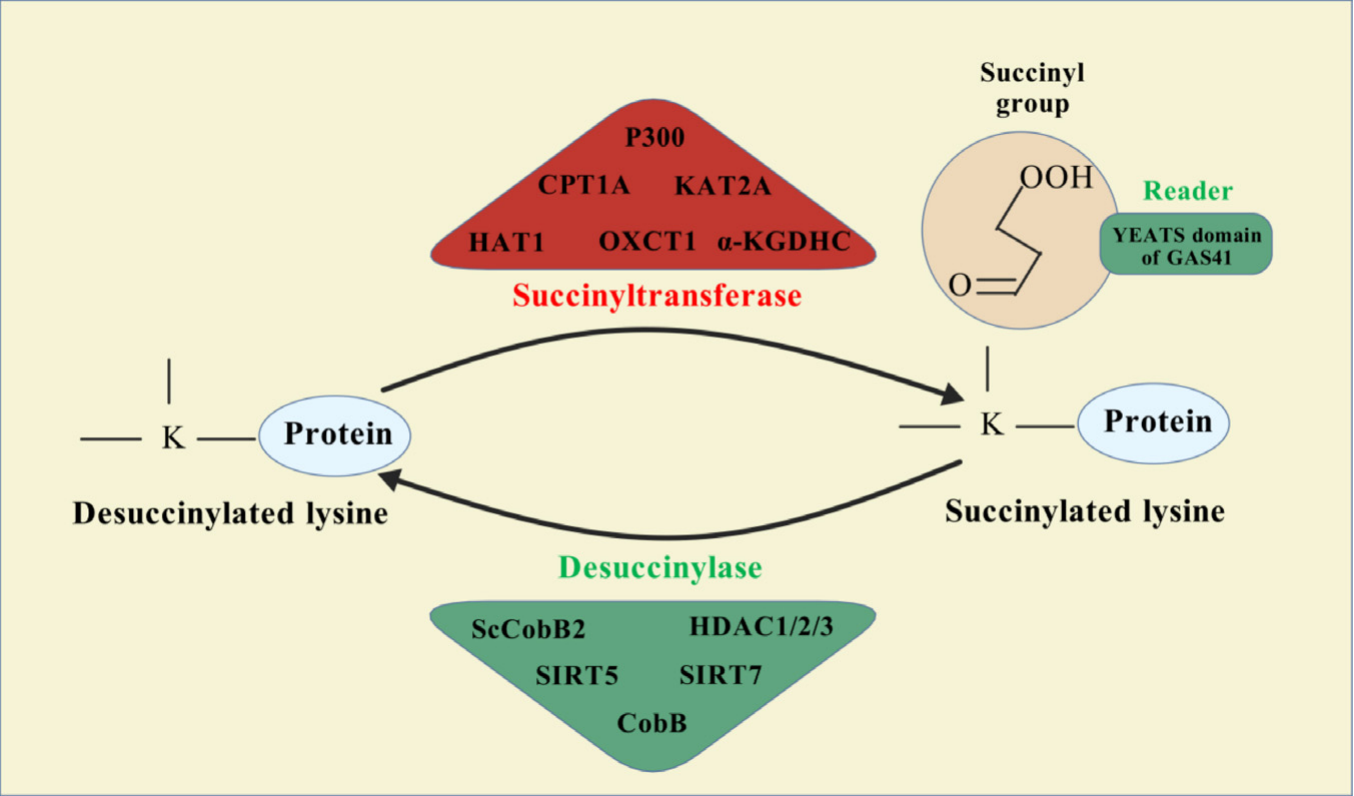
Succinylation modification mechanism diagram.

Protein PTMs represent a vast array of biochemical alterations that modulate protein function. To date, over 600 types of PTMs have been identified, including methylation, phosphorylation, ubiquitination, acetylation, succinylation, and lactylation (113). Among these, succinylation involves the enzymatic or non-enzymatic addition of a succinyl group to the ϵ-amino group of lysine residues within proteins, representing a reversible modification that can significantly influence protein spatial conformation, activity, stability, and intracellular localization (114). This acylation process can occur either non-enzymatically or enzymatically (16). Enzymatic succinylation primarily relies on succinyltransferases, which are analogous to histone acetyltransferases (HATs), facilitating the transfer of a succinyl group from succinyl-CoA to target protein lysine residues. This process is highly specific, allowing for precise regulation of protein function (115, 116) (**Figure 1**). In contrast, non-enzymatic succinylation occurs dynamically and widely in response to changes in the cellular metabolic environment, such as during metabolic or oxidative stress conditions. Under these circumstances, succinyl groups can directly bind to lysine residues through spontaneous chemical reactions. The dynamic equilibrium of this non-enzymatic modification is influenced by various factors, including the concentration of succinyl donors, intracellular pH levels, and redox status (116, 117).

Understanding the dynamic equilibrium of succinylation is critical for elucidating its role under both physiological and pathological conditions. Under normal physiological conditions, appropriate regulation of succinylation levels helps maintain cellular metabolic homeostasis and function. Conversely, in pathological states, aberrant increases or decreases in succinylation can lead to cellular dysfunction, thereby promoting disease progression. For instance, in cancer cells, succinylation influences cell proliferation and apoptosis by modulating the activity of key metabolic enzymes (116, 118). Moreover, succinylation is closely associated with the development and progression of various metabolic diseases, including liver metabolism disorders and diabetes. The main factors affecting the dynamic balance of succinylation include cellular metabolic status, environmental conditions (such as temperature and pH), and cellular signaling pathways (119, 120). Therefore, delving deeper into the regulatory mechanisms governing succinylation’s dynamic balance could provide new insights and potential therapeutic targets for preventing and treating related diseases.

Lysine succinylation is a prevalent PTM observed in both eukaryotic and prokaryotic cells (121), with its role in eukaryotic cells being closely associated with cancer progression. Within the nucleus of eukaryotic cells, lysine succinylation has been identified at specific histone sites, including H1.3K65, H1.3K86, H2BK109, H2BK117, H3K79, H3K120, and H3K122 (122). Additionally, non-histone proteins such as HMGB2K114, HMGB1K127, HMGB1K114, and HMGB1K43 also undergo succinylation (123). In the cytoplasm, lysine succinylation affects a wide array of metabolic enzymes and regulatory proteins. These include glycolytic enzymes and those involved in the TCA cycle, such as PDK3, IDH2, ACO2, DLAT, PDHA1, PITRM1, GOT2, MDH2, IDH3B, and SDHA. Enzymes involved in fatty acid metabolism, like ACAA2, HSD17B10, ETFa, HADHB, and HADHA, are also subject to this PTM. Proteins participating in ketone body metabolism, such as OXCT1 and ACAT1, and those related to reactive oxygen species (ROS) scavenging, including SOD, PRX, and GPX, exhibit succinylation as well (124). Thus, in eukaryotes, the dynamic regulation of protein succinylation and desuccinylation modulates various cellular processes, including metabolism, transcriptional regulation, and DNA damage repair. These processes are essential for maintaining normal cellular functions and are intimately linked with the occurrence and development of tumors.

### 3.2 The role of succinylation in energy metabolism, oxidative stress response, and gene expression regulation

Succinyl-coenzyme A (succinyl-CoA) serves as the primary donor of succinyl groups, primarily derived from the mitochondrial TCA cycle through the oxidative decarboxylation of α-ketoglutarate. Beyond glucose metabolism, the catabolism of amino acids such as methionine, threonine, valine, and isoleucine can also generate succinyl-CoA. Additionally, short-chain fatty acid ω-oxidation products, including hydroxy fatty acids, can be converted into succinyl-CoA. For instance, fibroblasts have been shown to utilize fatty acids as an effective source of succinyl-CoA under conditions where glucose and pyruvate are absent from the culture medium (125). The intracellular concentration of succinyl-CoA directly influences the level of succinylation modification (126), indicating that alterations in metabolic pathways can lead to corresponding changes in protein succinylation.

In the realm of energy metabolism, succinylation plays a pivotal role in modulating the activity of key metabolic enzymes. Studies have demonstrated that succinylation can influence the function of TCA cycle enzymes, thereby affecting overall mitochondrial energy production. This modification is particularly relevant in conditions characterized by mitochondrial dysfunction, such as heart failure and ischemic stroke. In models of heart failure, altered succinylation patterns have been linked to impaired oxidative phosphorylation capacity and dysregulated energy metabolism, highlighting the potential of targeting succinylation pathways for therapeutic interventions (127). The dynamic regulation of succinylation, mediated by enzymes such as SIRT5, which desuccinylates metabolic enzymes, underscores the complexity of metabolic regulation and its impact on cellular energy homeostasis (17). SIRT5’s role in controlling succinylation levels further emphasizes the intricate balance required for maintaining optimal metabolic function.

Oxidative stress represents another critical domain where succinylation exerts its regulatory influence. Accumulation of ROS can lead to oxidative damage, which is implicated in various diseases, including neurodegenerative disorders and cardiovascular diseases. Recent evidence suggests that succinylation can modulate ROS generation by influencing the stability and activity of antioxidant enzymes. For instance, succinylation of specific proteins involved in the antioxidant response can either enhance or inhibit their activity, thereby shaping the cellular response to oxidative stress (128). This regulatory mechanism is particularly significant in the context of aging and neuroinflammation, where increased succinylation levels have been associated with a senescence phenotype in microglia, suggesting a potential link between succinylation and age-related neurodegenerative processes (129).

Succinylation also plays a substantial role in regulating gene expression. This modification can alter chromatin structure and transcription factor activity, thereby influencing the transcription of genes involved in metabolism, stress response, and other critical cellular functions. Notably, succinylation of histones has been implicated in the regulation of gene expression patterns associated with tumorigenesis, indicating that this PTM could serve as a potential therapeutic target in cancer (130). Moreover, the interplay between succinylation and other PTMs, such as acetylation and phosphorylation, adds an additional layer of complexity to the regulatory networks governing gene expression and cellular responses to environmental cues (131).

In summary, succinylation emerges as a crucial post-translational modification that intricately links energy metabolism, oxidative stress response, and gene expression regulation. Ongoing research into the mechanisms and effects of succinylation continues to unveil its significance in maintaining cellular homeostasis and its potential implications in various diseases. As our understanding of succinylation deepens, it holds promise as a target for therapeutic interventions aimed at modulating metabolic disorders, oxidative stress-related conditions, and cancer (17).

### 3.3 Mechanism of protein desuccinylation catalyzed by SIRT5

Succinylation modification plays an essential role in various biological processes, and the regulatory mechanism of SIRT5 on succinylation has garnered increasing attention. Advances in the study of the deacylase SIRT5 have confirmed its dual capabilities: not only does it function as a deacetylase but it also exhibits potent desuccinylase activity (27). This versatility positions SIRT5 as a key regulator of cellular metabolism and other critical biological functions.

The enzymatic process by which SIRT5 catalyzes protein desuccinylation involves several critical steps that reflect the general mechanism of NAD+-dependent deacylation enzymes. Initially, SIRT5 must recognize and bind to the succinylated protein substrate. This binding typically occurs through the identification of specific sequence motifs or structural features on the substrate, particularly those harboring succinylated lysine residues. Upon binding, SIRT5 utilizes NAD+ as a covalent catalyst. In this context, NAD+ serves both as an electron donor and as a component that generates a covalent intermediate (ADP-ribose) during the reaction—a step essential for deacylation. The active site of SIRT5 contains a conserved cysteine residue that forms a covalent bond with the succinyl group of the succinyl-lysine residue. During this interaction, NAD+ is converted into nicotinamide and released from the complex. Subsequently, SIRT5 undergoes a series of chemical rearrangements to transfer the succinyl group from the substrate to the cysteine residue within the enzyme’s active site, forming a succinyl-enzyme intermediate. Finally, a water molecule attacks the succinyl group within the succinyl-enzyme intermediate, leading to the cleavage of the succinyl group. This results in the release of free succinate and the restoration of the unmodified state of the protein substrate. Once the reaction is complete, the desuccinylated protein is released from SIRT5, and the enzyme is reset to engage with another succinylated substrate, continuing the desuccinylation cycle (132). Through these catalytic steps, SIRT5 effectively removes succinyl groups from proteins, restoring the original state of lysine residues or altering their chemical environment. This action influences protein function, stability, or interactions with other molecules. Such deacylation is crucial for cellular metabolism, signaling pathways [such as NF-κB and IRF signaling (133), Notch and β-catenin signaling (134)], and adaptation to environmental changes.

The desuccinylation process mediated by SIRT5 is vital for regulating various cellular functions, including metabolism and stress responses. By modulating the succinylation state of proteins, SIRT5 can influence metabolic pathways, transcriptional activities, and other cellular processes, thereby playing a significant role in health and disease, including cancer development and progression.

### 3.4 The comparison of SIRT5 with the other desuccinylase

**Figure 2:**
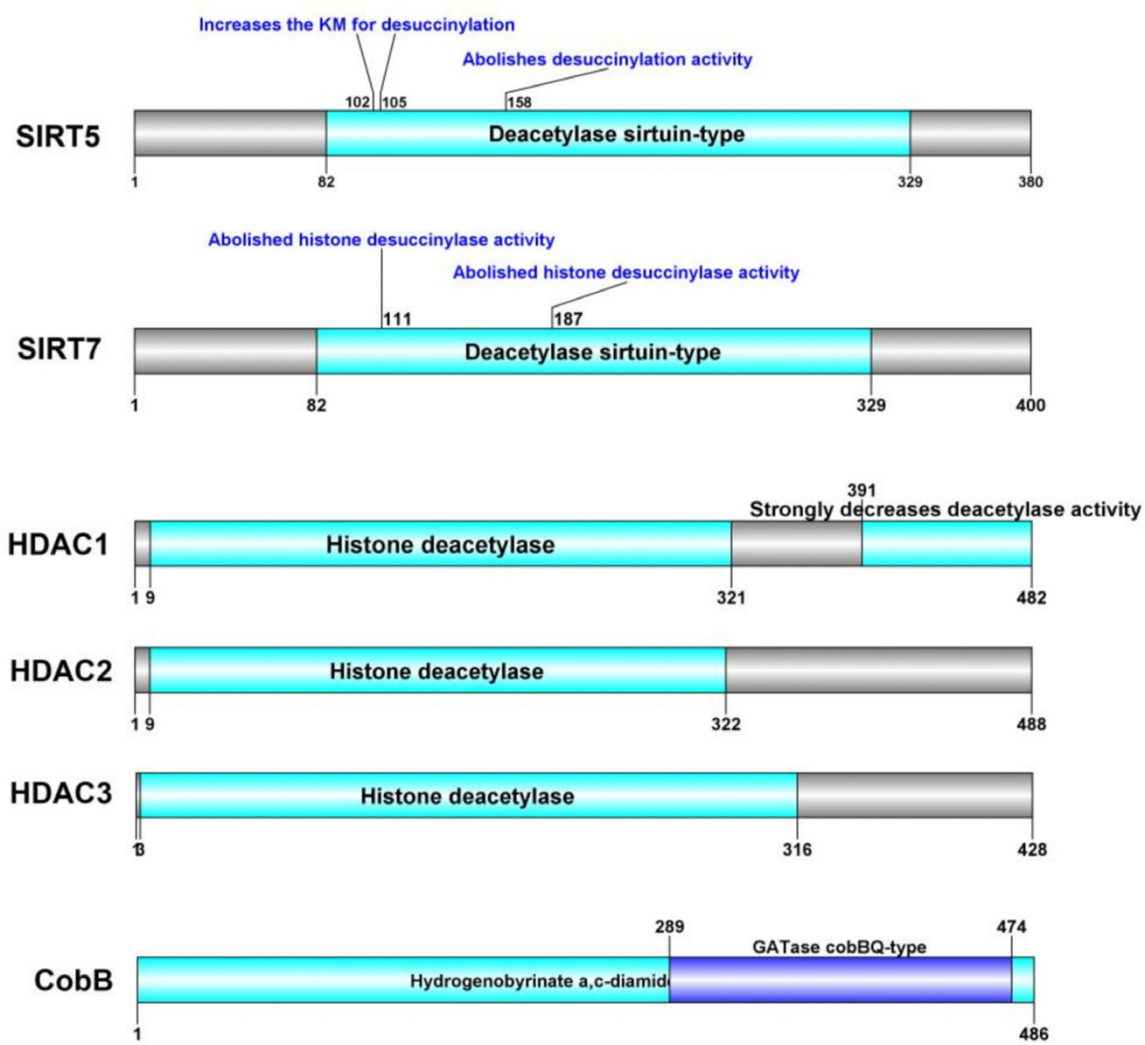
Succinylation modification specific cite of desuccinylase.

In addition to the well-characterized SIRT5 and SIRT7, recent studies have identified new proteins that exhibit desuccinylase activity. Notably, Jialun Li et al. (135) reported that histone desuccinylation is predominantly catalyzed by class I histone deacetylases (HDAC1/2/3). Inhibition or depletion of HDAC1/2/3 resulted in a significant increase in global histone succinylation levels, while ectopic expression of these enzymes—but not their deacetylase-inactive mutants—reduced global histone succinylation. Furthermore, *in vitro* assays demonstrated robust histone desuccinylase activity for class I HDAC1/2/3 complexes. These findings establish that class I HDAC1/2/3, rather than SIRT family proteins, are the principal histone desuccinylases, particularly important for promoter histone desuccinylation. The understanding of desuccinylation mechanisms in microorganisms remains in its infancy due to the paucity of identified specific desuccinylases. CobB, a known Sir2-like bacterial lysine deacetylase, was recently identified as the first prokaryotic enzyme with desuccinylation activity (136). The characterization of CobB as a bifunctional enzyme capable of both lysine desuccinylation and deacetylation suggests that eukaryotic Kac-regulatory enzymes may possess enzymatic activities on various lysine acylations with distinct structures. Additionally, in the model soil bacterium Streptomyces coelicolor, a sirtuin-like protein named ScCobB2 was biochemically characterized as a divergent desuccinylase. Comparative LC-MS/MS analysis of the ΔScCobB2 mutant versus wild-type succinylome revealed a total of 673 unique succinylated sites, with 470 sites quantified across 317 proteins. Further quantitative analysis indicated that at least 114 proteins involved in two major pathways—protein biosynthesis and carbon metabolism—are markedly hypersuccinylated in ΔScCobB2 cells (137). We conducted an analysis of the protein domains of these desuccinylases. **Figure 2** illustrates the specific sites regulated by SIRT5 and SIRT7 in the context of succinylation modification.

In summary, while SIRT5 is well-characterized as a desuccinylase with distinctive features, the existence and characteristics of other desuccinylases remain speculative without further research. SIRT5 stands out due to its defined role in mitochondrial function and its dependence on NAD+, distinguishing it from potential non-sirtuin desuccinylases that might operate through different mechanisms and within distinct cellular contexts. Future investigations into the diversity and specificity of desuccinylases will be crucial for elucidating their roles in cellular regulation and disease pathogenesis.

### 3.5 Factors influencing SIRT5 activity

Given the unique properties and functions of SIRT5 discussed previously, this section delves into the primary factors that influence its activity. As an NAD+-dependent deacylase, the intracellular concentration of NAD+ is crucial for SIRT5’s functionality. NAD+ serves dual roles: it acts as a cofactor essential for SIRT5’s desuccinylation reaction and operates as a rate-limiting factor for enzyme activity. The catalytic mechanism of SIRT5 requires NAD+ as a cofactor. During catalysis, NAD+ is consumed, generating nicotinamide and an ADP-ribose moiety. This process is pivotal for forming a covalent intermediate necessary for the desuccinylation reaction. The active site of SIRT5 contains a conserved cysteine residue that forms a covalent bond with the succinyl group of succinyl-lysine. Simultaneously, the ADP-ribose portion of NAD+ transiently forms a covalent complex with the enzyme, facilitating subsequent hydrolysis steps (8). Variations in intracellular NAD+ levels directly impact SIRT5 activity. Higher NAD+ levels enhance SIRT5’s desuccinylase activity by providing sufficient cofactor support for the catalytic reaction, while decreased NAD+ levels reduce SIRT5 activity due to insufficient cofactor availability (138). The cellular metabolic state, particularly the energy status, significantly influences NAD+ levels (139). For instance, under conditions of fasting or caloric restriction, NAD+ levels increase, potentially enhancing SIRT5 activity (140). Conversely, in states characterized by high-fat diets or obesity, NAD+ levels may decrease, leading to reduced SIRT5 activity (141). The ratio of NAD+ to reduced nicotinamide adenine dinucleotide (NADH) also plays a critical role in determining SIRT5 activity. A higher NAD+/NADH ratio generally promotes SIRT5 activity, indicating greater availability of NAD+ as a cofactor, whereas a lower ratio can inhibit SIRT5 activity (121). During stress responses, such as oxidative stress or hypoxia, cells experience fluctuations in NAD+ levels, which directly affect SIRT5 activity and consequently influence the cellular response to these stress conditions (142, 143). In certain disease states, including diabetes and cardiovascular diseases, alterations in NAD+ levels can indirectly impact SIRT5 activity and its regulatory role in cellular metabolism and signaling (142, 144). Changes in cellular metabolic status directly influence the supply of NAD+, thereby affecting SIRT5 activity.

Understanding the impact of NAD+ levels on SIRT5 activity can provide insights into the enzyme’s role in cellular physiology and offer potential intervention strategies for related diseases, including cancer.

## 4 Role of SIRT5 in cancer

**Figure 3:**
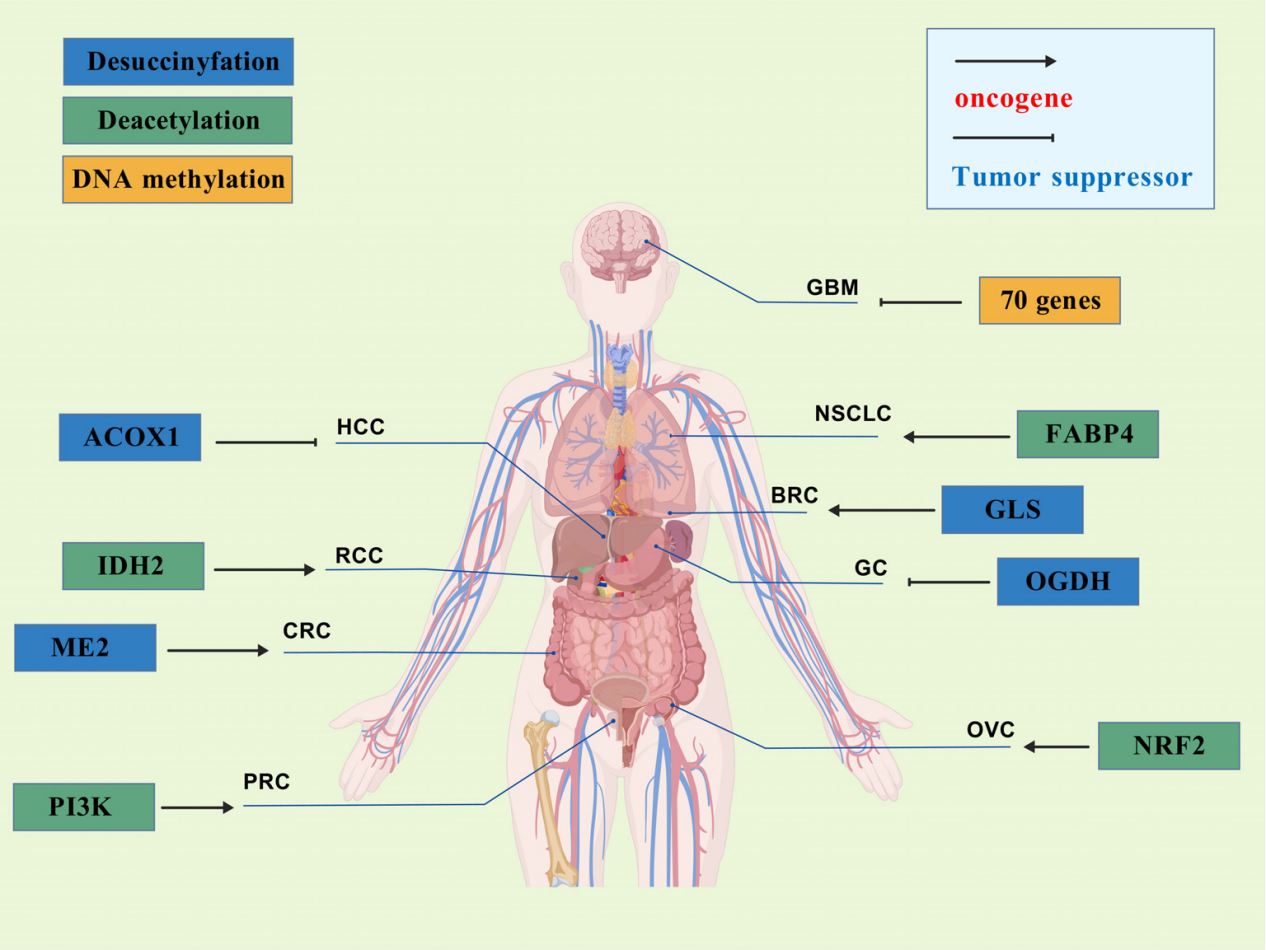
The role of SIRT5 in different cancers.

Succinylation modifications have been implicated in various malignancies, including lung cancer, melanoma, hepatocellular carcinoma, osteosarcoma, neurologic malignancies, renal cell carcinoma, thyroid cancer, and colorectal cancer (119, 145, 146). However, the role of succinylation in tumor progression is contingent upon the specific succinylation-modified genes, which can exert either tumor-suppressive or oncogenic effects. SIRT5, identified as the latest desuccinylation gene, acts as a double-edged sword in tumorigenesis. By modulating the expression of different target genes, SIRT5 can either inhibit or promote tumor development. The specific regulatory effects of SIRT5 on different cancer cells behaviors were shown in **Table 2**, **Figure 3**.

**Table 2:** The role of SIRT5 in different cancers.

Cancer type	Function	Functional involvement	Target gene	Protein modification
Colorectal cancer (147)	oncogene	Mitochondrial respiration and proliferation	ME2	Desuccinylation
Breast cancer (148)	oncogene	Glutamine metabolism and proliferation	GLS	Desuccinylation
Non-small cell lung cancer (95)	oncogene	Tumor growth	FABP4	Deacetylation
Ovarian cancer (149)	oncogene	Cell growth and cisplatin-resistance	NRF2	Deacetylation
Renal cancer (150)	oncogene	Sunitinib‐resistant mitochondrial functions and antioxidant capacity	IDH2	Deacetylation
Hepatocellular carcinoma (97)	Tumor suppressor	H2O2 production and oxidative DNA damage	ACOX1	Desuccinylation
Gastric cancer (151)	Tumor suppressor	cell growth, migration, mitochondrial functions and redox status.	OGDH	Desuccinylation
Prostate cancer (152)	Tumor suppressor	Metastasis	PI3K	Deacetylation
Glioblastoma (153)	Tumor suppressor	Occurrence and prognosis	70 differently expressed genes	DNA methylation

ME2, malic enzyme 2; GLS, glutaminase; FABP4, fatty acid binding protein 4; NRF2, nuclear factor erythroid 2-related factor 2; IDH2, isocitrate dehydrogenase 2; ACOX1, acyl-CoA oxidase 1; OGDH, oxoglutarate dehydrogenase; PI3K, phosphatidylinositol-4,5-bisphosphate 3-kinase.

### 4.1 SIRT5-mediated succinylation regulation and its role in suppressing tumor progression

Studies have demonstrated that the succinylation of superoxide dismutase 1 (SOD1) promotes cancer cell proliferation; however, SIRT5 can reverse this effect by mediating desuccinylation and thereby restoring SOD1 enzyme activity. Lung cancer cells with mutations at the succinylation sites of SOD1 exhibit significantly reduced proliferation rates, indicating the tumor-suppressive role of SIRT5 (154). Clark et al. (155) found that mutant isocitrate dehydrogenase 1 (IDH1) converts α-ketoglutarate (α-KG) into R-2-hydroxyglutarate (R-2-HG), an α-KG analog that elevates succinyl-CoA levels, leading to abnormal succinylation of mitochondrial proteins and promoting cancer cell growth while impairing apoptosis. However, ectopic expression of SIRT5 reverses the metabolic defects and apoptotic resistance in IDH1-mutated glioma cells, impairing their growth both *in vitro* and *in vivo* (156). In hepatocellular carcinoma (HCC), SIRT5 also exerts a tumor-suppressive function. Expression of SIRT5 is lower in primary liver cancer tissues compared to normal tissues. Aberrant activation of acyl-CoA oxidase 1 (ACOX1), which is involved in H_2_O_2_ generation, leads to DNA oxidative damage and impaired liver function, contributing to HCC onset. SIRT5 can inhibit ACOX1 through desuccinylation, thereby reducing H_2_O_2_ levels and mitigating oxidative stress (97). In gastric cancer, Lu et al. (151) discovered that SIRT5 expression is significantly decreased in human gastric cancer tissues. Functional analyses indicated that overexpression of SIRT5 can inhibit gastric cancer cell growth both *in vitro* and *in vivo* by arresting the cell cycle at the G1/S phase and suppressing migration and invasion via modulation of epithelial-mesenchymal transition (EMT). Further analysis revealed that the tumor-suppressive effect of SIRT5 in gastric cancer is associated with the regulation of 2-oxoglutarate dehydrogenase (OGDH) expression. SIRT5-mediated desuccinylation of OGDH inhibits the OGDH complex’s activity, leading to reduced mitochondrial membrane potential, decreased ATP production, increased ROS levels, and altered NADP/NADPH ratios, ultimately suppressing gastric cancer progression. In prostate cancer, SIRT5 expression is significantly reduced, and a correlation between decreased SIRT5 levels and reduced patient survival has been established. Quantitative global succinylation profiling in prostate cancer revealed a significant increase in the succinylation of lysine 118 (K118su) of lactate dehydrogenase A (LDHA), enhancing LDH activity and exacerbating tumor progression. Overexpression of SIRT5 reduces LDHA-K118 succinylation, inhibiting the migration and invasion of prostate cancer cells and alleviating disease progression (157). Beyond these examples, SIRT5 has also been found to inhibit gastric cancer invasion by catalyzing the desuccinylation of S100A10 protein (158), and it can desuccinylate the K280 site of serine hydroxymethyltransferase 2 (SHMT2) protein, thereby inhibiting osteosarcoma development (159). Therefore, SIRT5 can inhibit tumor cell growth through interfering with multiple pathways.

### 4.2 SIRT5-mediated succinylation regulation promoting tumor initiation and progression

Recent studies have also identified SIRT5 as an oncogenic promoter through its involvement in various pathways across different malignancies. Teng et al. (147) discovered that mitochondrial malic enzyme 2 (ME2) is highly expressed in colorectal cancer (CRC) tissues, and knockdown of ME2 inhibits CRC cell proliferation. Further analysis revealed that overexpressed ME2 undergoes SIRT5-mediated desuccinylation. Deprivation of glutamine directly enhances the interaction between SIRT5 and ME2, promoting desuccinylation of ME2 at lysine 346 and thereby activating ME2 enzyme activity. This activation leads to increased cellular proliferation and tumor growth. In breast cancer, SIRT5 expression is significantly elevated, and knockout of SIRT5 can induce oxidative stress by increasing the succinylation of IDH2, leading to apoptosis in tumor tissues and inhibiting tumor growth (160). In RCC, SIRT5 interacts with subunit A of the succinate dehydrogenase complex (SDHA). Knockout of SIRT5 results in increased succinylation and expression levels of SDHA. Elevated SIRT5 expression has been observed in RCC cells and tissues, and SIRT5 knockout inhibits cancer cell proliferation. These findings suggest that SIRT5 promotes the occurrence and development of RCC by inhibiting SDHA succinylation (161). Thus, elevated SIRT5 expression has been observed in various cancers and correlates with poor patient prognosis.

Given the contrasting oncogenic and tumor-suppressive effects of SIRT5, it is evident that the specific role of SIRT5 depends on its key target genes and the type of tumor. Additionally, studies have found that the function of SIRT5 also depends on whether it performs desuccinylation or deacetylation (6). Currently, research on SIRT5 presents many unresolved questions. The investigation into SIRT5-mediated desuccinylation activity is still in its infancy, and the relationships and mechanisms between SIRT5 and multiple cancers require further exploration to provide guidance for future cancer treatments.

## 5 Potential of SIRT5 as a therapeutic target in cancers

The role of SIRT5-mediated desuccinylation in tumor progression underscores its potential as a therapeutic target for cancer treatment. In non-small cell lung cancer (NSCLC), quercetin has been shown to bind to SIRT5, thereby regulating SIRT5-mediated desuccinylation of PI3K. This interaction inhibits PI3K/AKT phosphorylation, subsequently blocking homologous recombination and non-homologous end-joining repair processes, leading to mitotic mutations and apoptosis, and ultimately alleviating NSCLC progression (162). In colorectal cancer (CRC), Zhang et al. (163) reported that extracellular vesicles isolated from Lactobacillus plantarum can inhibit SIRT5 expression, thus modulating the desuccinylation level of p53. This regulation leads to inhibition of CRC cell proliferation and glycolysis, effectively suppressing the *in vivo* growth of tumor tissues. Apart from pharmacological interventions, recent studies have identified upstream regulatory genes involved in tumor progression that affect SIRT5 expression. In hepatocellular carcinoma (HCC), Bai et al. (164) found that solute carrier family 25 member 20 (SLC25A51), a newly identified mammalian mitochondrial NAD+ transporter, is upregulated in human HCC specimens and cell lines. Further analysis revealed that SLC25A51 activates SIRT5 expression, promoting a metabolic shift from oxidative phosphorylation to glycolysis—a key mechanism driving tumor progression. Knockout of SLC25A51 reduces SIRT5 expression, thereby mitigating HCC progression. However, current research predominantly focuses on the oncogenic role of SIRT5, with limited information on enhancing its expression to exert tumor-suppressive effects. In gastric cancer, Tang et al. (165) demonstrated that SIRT5 expression is regulated by cyclin-dependent kinase 2 (CDK2). Typically, CDK2 exacerbates tumor progression by regulating cell cycle progression and DNA damage response. Conversely, knockout of CDK2 can inhibit malignant proliferation and aerobic glycolysis of cancer cells by increasing SIRT5 expression, revealing a novel role for SIRT5 as a tumor suppressor regulated by upstream genes in cancer.

The development of specific SIRT5 regulators has emerged as a promising approach in clinical cancer therapy. The information of specific SIRT5 regulators was showed in **Table 3**. Deng et al. (166) identified ϵ-N-thioglutaryl-lysine derivatives as potent inhibitors of SIRT5, with photo-crosslinking derivative 8 exhibiting the strongest inhibitory effect. Kinetic analysis revealed that these derivatives inhibit SIRT5 by competing with lysine substrates. Co-crystal structure analysis demonstrated that photo-crosslinking derivative 8 binds to SIRT5 via hydrogen bonds and electrostatic interactions with specific residues, occupying the lysine substrate binding site and potentially reacting with NAD+ to form a stable thio-intermediate. This structural insight provides valuable information for the design of drug-like inhibitors and cross-linking chemical probes for SIRT5-related research. Additionally, Jiang et al. (167) designed six N-terminal-to-side-chain cyclic tripeptides and evaluated their efficacy through *in vitro* deacetylase inhibition assays and proteolytic stability tests. Among these compounds, cyclic tripeptide 10 exhibited strong inhibition of SIRT5-mediated desuccinylation reactions and demonstrated superior proteolytic stability against SIRT5. Compared to previously reported potent and selective SIRT5 inhibitors, cyclic tripeptide 10 represents a novel modular scaffold, offering a new avenue for discovering improved SIRT5 inhibitors that could serve as chemical or pharmacological probes and potential treatments for tumors characterized by upregulated SIRT5-mediated desuccinylase activity. Regarding SIRT5 activators, MC3138, a selective SIRT5 activator, mimicked the effects of SIRT5 overexpression-mediated deacetylation and desuccinylation in pancreatic cancer cells, leading to reduced levels of metabolites such as glutamine and glutamate (168). Given that SIRT5 expression is downregulated in human and mouse pancreatic ductal adenocarcinomas, the application of MC3138 in pancreatic tumors showed inhibitory effects on proliferation. Combination treatment with gemcitabine may represent a therapeutic strategy for this type of cancer (169). Therefore, modulating SIRT5 expression appears to be an effective means of alleviating tumor progression. The ongoing development of small molecule inhibitors or activators of SIRT5 offers new strategies for future cancer treatments. As our understanding of SIRT5 functions deepens, its potential as a therapeutic target for cancer treatment becomes increasingly evident.

**Table 3:** The information of specific SIRT5 regulators.

Molecular name	Molecular structural formula	IC50
ϵ-N-thioglutaryl-lysine	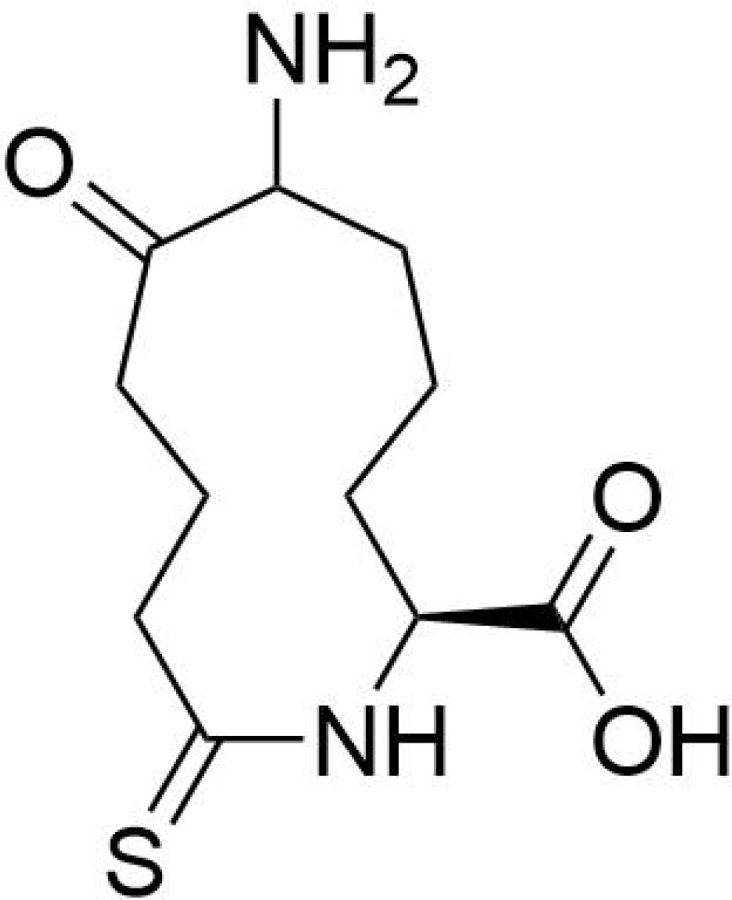	120 nM
N-terminal-to-side-chain cyclic tripeptides	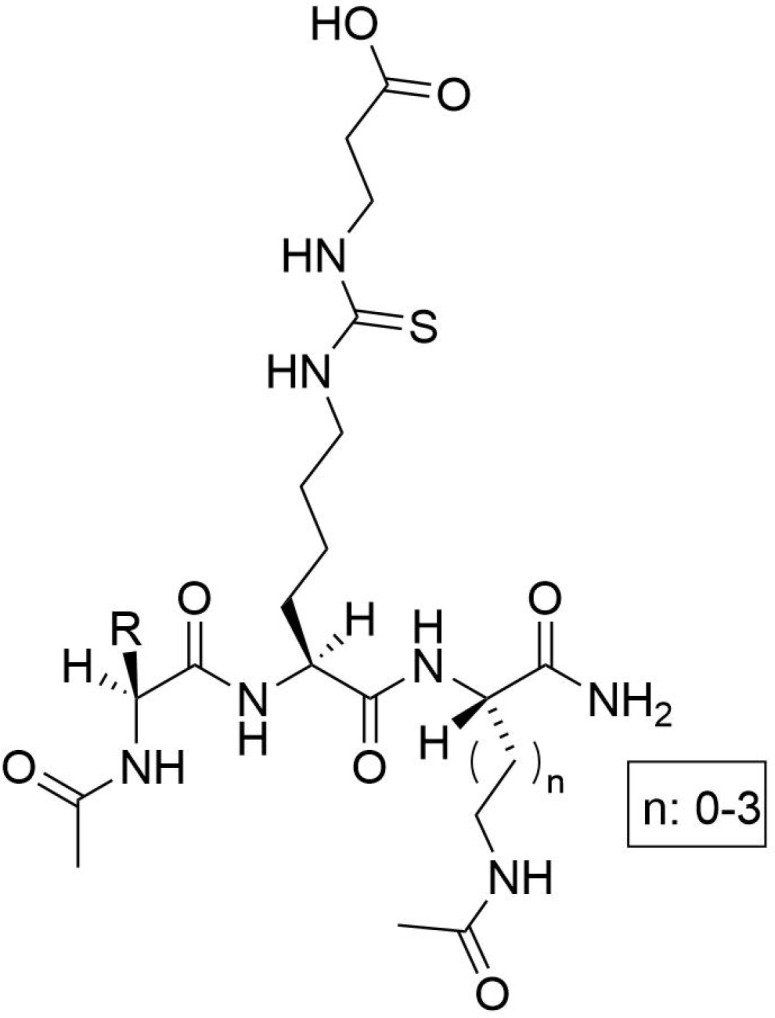	13.2 μM
cyclic tripeptide 10	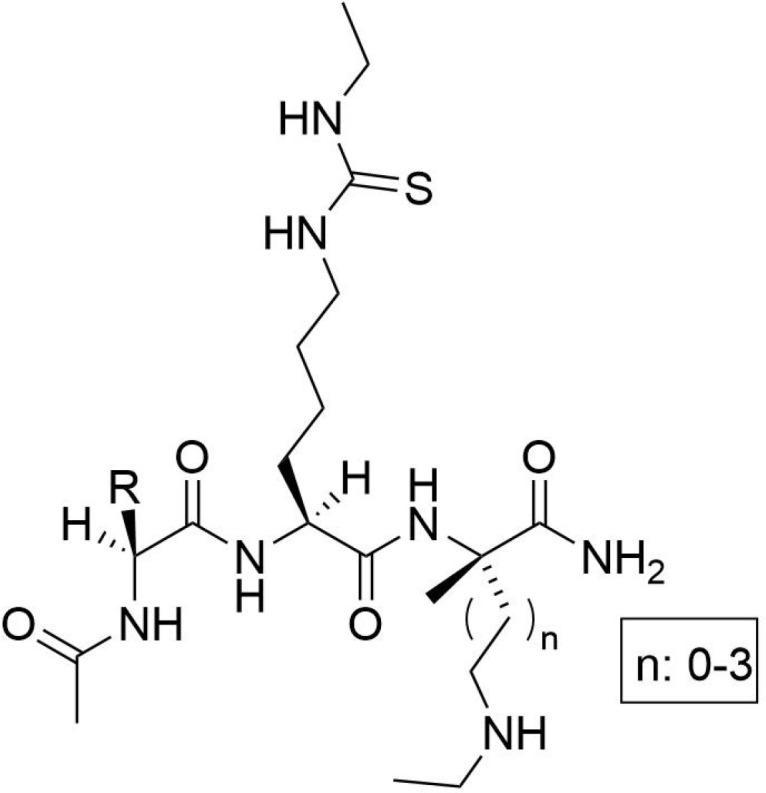	2.2 μM
gemcitabine	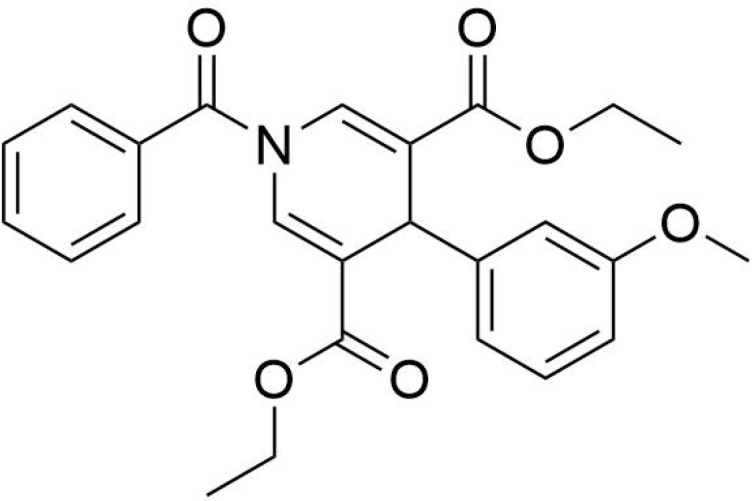	0.98 μmol/L,
MC3138	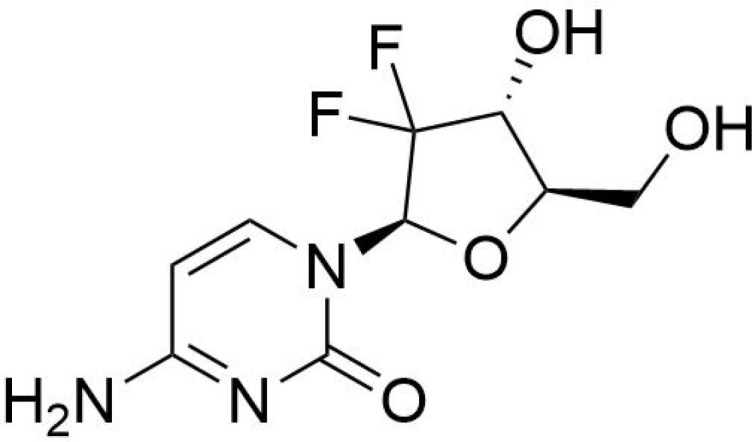	25.4 μM

## 6 Future research directions and challenges

Despite the promising role of SIRT5 in cancer therapy, several challenges and future research directions must be addressed. A primary challenge is the need for a deeper understanding of the molecular mechanisms by which SIRT5 exerts its effects on tumor biology. Although SIRT5 has been implicated in various pathways, its specific interactions and regulatory networks within different cancer types remain inadequately defined. Future studies should focus on elucidating these mechanisms, potentially utilizing advanced genomic and proteomic approaches to comprehensively map SIRT5 interactions within the tumor microenvironment (170). Moreover, the development of specific SIRT5 inhibitors or modulators is critical for translating these findings into clinical applications. This includes investigating the potential side effects and off-target effects of such therapies, as well as assessing their efficacy when used in combination with existing cancer treatments. It will also be important to explore dose-response relationships and pharmacokinetic properties to ensure optimal therapeutic outcomes. Additionally, exploring the role of SIRT5 in immune modulation could open new avenues for immunotherapy, particularly in cancers that exhibit resistance to current therapies (171). Understanding how SIRT5 influences immune cell function and tumor-immune interactions may provide insights into novel therapeutic strategies that combine SIRT5 modulation with immunotherapeutic approaches. As research progresses, addressing these challenges will be essential for harnessing the full therapeutic potential of SIRT5 in oncology. The integration of multi-disciplinary approaches, including systems biology, computational modeling, and translational research, will be crucial for overcoming the complexities associated with SIRT5’s multifaceted roles in cancer. Addressing these issues will not only enhance our understanding of SIRT5’s biological functions but also pave the way for innovative cancer therapies targeting this enzyme.

## 7 Conclusion

SIRT5, functioning primarily as a desuccinylase, exhibits significant regulatory roles in tumor biology. Advances in proteomics have led to the recognition that SIRT5 is not merely a deacetylase but increasingly serves as a critical desuccinylase involved in modulating multiple metabolic pathways, including glycolysis, the TCA cycle, fatty acid metabolism, and ROS scavenging. SIRT5 plays a pivotal role in cellular energy metabolism and homeostasis, with its dysregulation being implicated in various types of cancer. These findings underscore the central importance of SIRT5 in tumor metabolic reprogramming, suggesting that both SIRT5 itself and the succinylation modifications it regulates could serve as promising targets for the development of novel anticancer therapies. However, the specific functions of SIRT5 in different types of tumors remain to be further elucidated, particularly given its dual nature as a potential tumor suppressor in some contexts and a tumor promoter in others. Consequently, the application of SIRT5 as a therapeutic target necessitates personalized research approaches tailored to specific tumor types and microenvironments. Future studies should focus on delineating the precise mechanisms by which SIRT5 exerts its effects, considering the complex interplay between SIRT5 activity, metabolic alterations, and tumor progression. By addressing these challenges, researchers can harness the therapeutic potential of SIRT5 to develop more effective and targeted anticancer strategies.
